# The Diagnosis, Pathology, and Treatment of the Diseases of the Chest

**Published:** 1860-03

**Authors:** 


ART. III.—*The Diagnosis, Pathology, and Treatment of the Diseases of the Chest.* By W. W. GERHARD, M.D., one of the Physicians of the Pennsylvania Hospital, Fellow of the College of Physicians, Member of the American Philosophical Society, etc. Fourth edition, 8vo., pp. 448. J. B. Lippincott & Co.: Philadelphia, 1860.

IT affords us much pleasure to announce to our readers the appearance of this new edition of a work which has been so long and so favorably known in this country. The name of the distinguished author has been closely identified with the diagnosis, pathology, and treatment of the diseases of the chest for upwards of a quarter of a century, and we know of no one who is more thoroughly conversant with the subject than he. Educated in the School of Louis, Dr. Gerhard became early enamored with the study of thoracic diseases, and while yet quite a young man effected some very important discoveries which soon made his name extensively known both at home and abroad.

The work of Dr. Gerhard, originally published in lecture-form, comprises the results of a large clinical experience, and may be regarded as exhibiting a very full digest of the existing state of the department of science of which it treats. The present edition, as stated in the preface, has been thoroughly revised, carefully corrected, and much enlarged, about one hundred pages of new matter being introduced. Important additions have been made to the sections on pneumonia, phthisis, and cardiac diseases.

The author has devoted considerable space to the use of cod-liver oil in phthisis, and as his experience is large and valuable, we shall transfer here his principal conclusions respecting an article which has received so much attention during the last fifteen years in the treatment of this relentless malady.

Dr. Gerhard prefers altogether the light-colored oil, as it is better borne by the stomach, as well as more nutritious, than the brown oil. It is generally easily taken before meals, its most pleasant vehicle being milk, though its taste is most effectually disguised by the froth of porter. The existence of diarrhœa is no contra-indication to its exhibition. Its use increases the flesh, weight, and strength of the patient, but to be of any decidedly permanent benefit, the exhibition must be steadily persisted in for a long time.

"Within the last ten years, since these earlier observations upon cod-liver oil were made, my experience in its use has been very large, and enables me to speak positively on many points which were formerly somewhat doubtful.

"First. Is cod-liver oil as good a remedy in the management of phthisis as it was thought to be? My own settled conviction is, that the oil is one of the best nutrients that can be given; in that way it antagonizes phthisis to a certain degree, and sometimes puts a radical check on the disorder; but I do not believe that its properties depend upon any specific virtue. It is simply introducing into the system a large quantity of fatty matter, thus directly opposing the formation of tubercles. Indeed, I have sometimes thought that in those rare cases of phthisis in which there is little or no emaciation, cod-liver oil does not act so kindly as in those patients who assume the peculiarly thin and emaciated aspect of regular consumptives.

"Secondly. Cod-liver oil is not tolerated well during the hot months, in our climate. Patients are often obliged to abandon its use, or at any rate to restrict the quantity, during the summer; they may often resume it again when the weather becomes cool, and the powers of digestion are increased. The difficulty of taking it during the hot months depends entirely upon the extreme nausea which it then causes; if the patient does digest it well, he may cautiously continue it even in intense heat, but he should at once abandon it if his power to digest it becomes enfeebled.

"Thirdly. The remarks already made as to the use of the darker-colored oils have also been thoroughly confirmed. In Philadelphia, as in most other parts of the United States, there is now scarcely any other oil than the light-colored given—that is, either the livers of the fish are prepared very soon after they are obtained, or else the oil must be so purified as to destroy the offensive taste and aspect of the dark-colored variety.

"Fourthly. Patients can frequently continue the use of the oil for a long period; but after their stomachs have become thoroughly disgusted with the medicine, it will be found almost impossible for them to return to it. It is true that a temporary cessation of this remedy may cause the stomachs of the patients to be so much relieved that they can resume its use, but this resumption is but temporary. They are rarely able to return to it again as a permanent plan of treatment, after they have once been completely disgusted with it. Hence I rarely attempt to persist in its administration when unequivocal evidence of disgust on the part of the patient is exhibited.

"Fifthly. The employment of cod-liver oil has to a certain degree diminished. Physicians who resorted to it under the conviction that it was a sure remedy for phthisis have been disappointed in the results obtained from it. Many of them therefore have very much abandoned this medicine; the error with them however is, that they have expected too much from it, and finding themselves to a certain degree deceived, they have neglected a valuable remedy. The worth of it remains, although this was exaggerated by the overweening expectations of its early advocates."

Of the use of the different phosphatic salts, first proposed by Dr. Churchill, now of Paris, in the treatment of phthisis, the opinion of our author coincides with that of nearly all the more experienced and trustworthy practitioners of this and other countries. We have long been satisfied that their value was chiefly imaginary, an undue importance having been attached to these remedies by the fact that they have received the sanction of a few distinguished physicians on this side of the Atlantic, whose influence has been sufficient to turn the heads of many of the lesser lights of the profession. But we will allow the author to speak for himself.

"Another mode of treating phthisis which has attracted much notice within the last few years is the use of different phosphatic salts, given in connection with one another. These are the phospates of iron, soda, lime, and potash. This plan was proposed by Dr. Churchill, and was founded on the supposition that the addition of these salts to the blood would enable it to withstand the tendency to tuberculous deposit. I have been chiefly in the habit of giving these salts simply mixed with water, directing the patient to shake it up before taking it, so as to suspend the phosphate of iron, which is of course insoluble. The mixture is made according to the following formula:—

"℞.—Sodæ phospat, ʒiv; Calcis phosphat, ʒij; Potassæ phosphat, ʒi; Ferri phosphat, ℈ij; Aq. Rosæ, ℥vi.—M.

"S. A teaspoonful after breakfast and dinner, first shaking the bottle. If the substance is well shaken, the phosphate of iron may be of course sufficiently suspended to be taken in a proper dose.

"Mucilage should not be added, as it would soon spoil. My friend Dr. Jackson has been in the habit of giving the same medicine in syrup, making a more uniform and prettier preparation, but one which I do not like so well as a simple suspension of these salts. If, however, this preparation should be preferred, the best mode of making it is one given by Mr. Parrish in the Am. Journ. of Pharmacy for 1857, vol. xxix. p. 572.

"The use of these phosphates became very general in Philadelphia, but has much diminished within the past year or two. This comparative disuse of them has arisen simply from the doubt that remained in the minds of those who employed them as to their utility. The iron produces, of course, its usual effect; but whether the phosphoric acid is really taken into the blood, and deposited in quantities sufficient to cause the absorption of tubercles, or their conversion into calcareous matter, seems to me very doubtful. It has been also supposed that by absorption the phosphorus is diffused throughout the whole body, and to a certain degree antagonizes the process of emaciation. I have never been able to convince myself with certainty that this good result had occurred; and although I still occasionally use the preparation, I do so with a doubt whether any good effects follow from it."

With these extracts and remarks we close the book before us, regretting that our limits will not permit us to indulge in further comments. The work is one of sterling value, and no American library can be complete without it.

